# Prevalence of clinical‐level emotional/behavioral problems in schoolchildren during the coronavirus disease 2019 pandemic in Japan: A prospective cohort study

**DOI:** 10.1111/jcv2.12007

**Published:** 2021-04-28

**Authors:** Fumito Takahashi, Hideo Honda

**Affiliations:** ^1^ Institute of Education Shinshu University Nagano Japan; ^2^ Department of Child and Adolescent Developmental Psychiatry Shinshu University School of Medicine Matsumoto Japan

**Keywords:** adolescents, children, COVID‐19, hyperactivity, inattention, mental health, school closure

## Abstract

**Background:**

Several empirical studies have investigated negative mental health outcomes related to the spread of infectious diseases, including coronavirus disease 2019 (COVID‐19). However, little is known about children's emotional/behavioral problems, especially externalizing problems, during such situations. This prospective cohort study aimed to investigate pandemic‐related emotional/behavioral problems and their risk factors among schoolchildren in Japan.

**Methods:**

A total of 4800 parents with children in grades 1−12 participated in a two‐wave longitudinal survey. Wave 1 and Wave 2 were conducted on March 4−8 and May 15−18, 2020, respectively. Survey items included demographic information, parental depression, children's diagnoses of neurodevelopmental disorders, the total length of school closure, and emotional/behavioral problems. Children's emotional/behavioral problems were assessed using the Strengths and Difficulties Questionnaire (SDQ), which has cutoff points to differentiate clinical‐level problems, which were the primary focus of this study.

**Results:**

The proportions of clinical‐level problems were higher at Wave 2 (emotional symptoms = 24.8%, conduct problems = 22.7%, hyperactivity/inattention = 36.8%, peer relationship problems = 36.2%, and lack of prosocial behavior = 23.5%) compared to Wave 1. Lower grade‐level and lower annual family income predicted the increased proportions of children's clinical‐level emotional symptoms, hyperactivity/inattention, and prosocial behavior at Wave 2. The total length of school closure was not a significant predictor of subsequent emotional/behavioral problems. The highest proportion of clinical‐level problems at Wave 2 for the four SDQ subscales was observed in children with neurodevelopmental disorders.

**Conclusions:**

The number of schoolchildren with severe emotional/behavioral problems increased during the COVID‐19 pandemic. Appropriate prevention and early intervention programs should be provided, especially for children who are in lower grade levels, have low family incomes, or have neurodevelopmental disorders.

## Graphical abstract

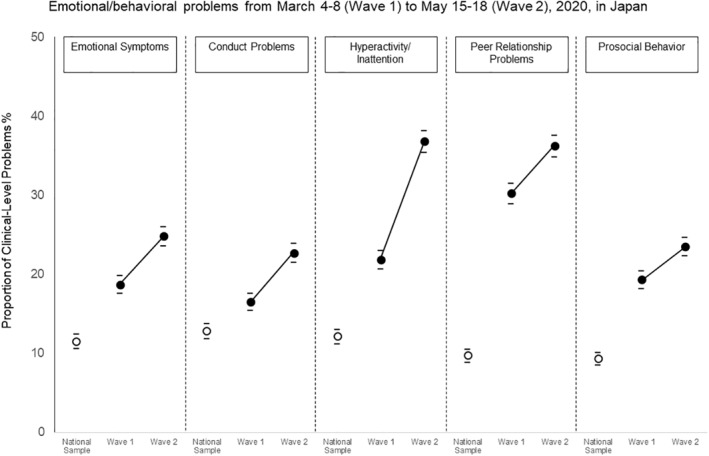

**Key points**

- Several studies reported an association between the COVID‐19 pandemic and higher levels of internalizing problems, such as depression and anxiety, in the general population.
- In addition to internalizing problems, children exhibited more externalizing problems—hyperactivity/inattention—and less prosocial behavior in May than in March 2020.
- Increases in emotional/behavioral problems were predicted by lower school grade and lower family income.
- The highest proportion of clinical‐level emotional/behavioral problems were observed in children with neurodevelopmental disorders.
- Appropriate prevention and early intervention programs should be provided, especially for children who are in lower grade levels, have low family incomes, or have neurodevelopmental disorders, during the COVID‐19 pandemic.

## INTRODUCTION

Coronavirus disease 2019 (COVID‐19) has had a tremendous impact on daily life for people worldwide, and there are concerns about its impact on mental as well as physical health. Several empirical studies have investigated the mental health consequences of the spread of infectious diseases, including COVID‐19, in general adult populations (Pierce et al., 2020; Wang et al., 2020; Wang, Pan, Wan, Tan, Xu, McIntyre, et al., 2020), suspected or confirmed patients (Jeong et al., 2016; Mak et al., 2009), and healthcare workers (Lai et al., 2020; Rossi et al., 2020). Although the number of COVID‐19 cases in Japan is relatively low (i.e., 16,305 confirmed cases out of 124 million Japanese citizens as of May 18, 2020), there are still concerns about the pandemic's substantial impact on mental health in the general population (Shigemura et al., 2020). Given this situation, many researchers have emphasized mental health assessment, prevention, and treatment in the context of a pandemic (Bao et al., 2020; Torales et al., 2020).

Research on the impact of COVID‐19 on children and adolescents has also focused on mental illnesses (Golberstein et al., 2020; Racine et al., 2020) in addition to physical symptoms (Ding et al., 2020). One study reported higher levels of depression and anxiety in samples obtained during the COVID‐19 pandemic than samples from before the pandemic (Xie et al., 2020). Subsequently, two cross‐sectional studies that were conducted during the pandemic reported that 43.7%−44.5% and 37.4%−38.0% of children experienced mild to severe depression and anxiety, respectively (Qi et al., 2020; Zhou et al., 2020). Regarding behavioral problems, two studies reported that parents of children with neurodevelopmental disorders felt their children's behavioral problems worsened during the COVID‐19 pandemic (Colizzi et al., 2020; Zhang et al., 2020). These findings indicate that children's emotional/behavioral problems during a pandemic may be an essential topic to be addressed and needs further research. An overview of literature regarding mental health issues in children and adolescents during epidemics is presented in Table S1.

Despite many previous research efforts, there are three problems that need to be addressed to gain a better understanding of child and adolescent emotional/behavioral problems during the pandemic. First, since all previous studies were cross‐sectional and have described between group differences of the reference and study samples, there is no direct evidence of within‐group changes in children's emotional/behavioral problems during the spread of COVID‐19. Some prospective longitudinal surveys have been conducted targeting adult populations (e.g., Pierce et al., 2020; Wang, Pan, Wan, Tan, Xu, McIntyre, et al., 2020); however, no such studies have been conducted with children or adolescents. Therefore, the same research design should be applied when investigating the effect of COVID‐19 on emotional/behavioral problems in schoolchildren.

Second, compared to internalizing problems such as depression and anxiety (Courtney et al., 2020), little attention has been paid to externalizing problems, such as conduct problems and hyperactivity, during the COVID‐19 pandemic. Although two retrospective studies reported increased behavioral problems in children with autism spectrum disorder (Colizzi et al., 2020) and attention‐deficit/hyperactivity disorder (Zhang et al., 2020), these studies used original but invalidated items for measuring externalizing problems. As noted by Jefsen et al. (2020), a thorough characterization of pandemic‐related psychopathology based on well‐validated measures is crucial for making progress on evidence‐based policy and clinical practice.

Third, little is known about the possible risk factors of emotional/behavioral problems during a pandemic such as COVID‐19 (Jefsen et al., 2020). In addition to the general risk factors, such as low family income or children's neurodevelopmental disorders, one critical concern related to schoolchildren's emotional/behavioral problems is the impact of school closures (McGinty et al., 2020; Viner et al., 2020). School closures may deprive children of access to free lunches, clean water, social relationships, adequate physical activity, and other resources essential for children's physical and mental health (Lancet Child Adolescent Health, 2020). It is believed that the longer the school closure is, the stronger the effect on children and adolescents.

The purpose of the current longitudinal study was to investigate whether the emotional/behavioral problems in schoolchildren increased during the rapid spread of COVID‐19 and, if so, identify the possible risk factors related to such problems. It was hypothesized that (a) more children would exhibit clinical‐level emotional/behavioral problems as the pandemic persists, and (b) exposure to prolonged school closure would predict emotional/behavioral problems in children and adolescents.

## MATERIALS AND METHODS

### Ethical considerations

The Shinshu University Ethics Committee on Educational Research approved the current study's procedures; all the procedures conformed with local legal requirements. All study participants provided informed consent to participate in this survey.

### Participants and procedure

The participants were drawn from members registered with a Japanese online survey company, which is one of the largest research companies nationwide with more than 2.2 million members. All study participants provided informed consent, completed the survey, and received around 100 Japanese Yen (approximately 0.9 US Dollars, 0.7 British Pounds, or 0.8 euros) as an incentive for their participation.

In the current two‐wave prospective cohort study, a staged sampling of participants was conducted. Initially, parents with children in grades 1−12 (ages 6−18 years) were selected and sent an invitation to participate in the Wave 1 survey in March 2020. Twenty‐four segments (i.e., boys and girls, grades 1−12) were then created, and data were collected until each segment reached 200 participants (i.e., 200 first‐grade boys, 200 first‐grade girls, …, 200 twelfth‐grade boys, and 200 twelfth‐grade girls). As a result, the Wave 1 survey yielded a total sample of 4800 families. Respondents were asked to answer questions regarding their firstborn child. All participants in the Wave 1 survey were invited to participate in the Wave 2 survey in May 2020, and 3847 respondents (80.1%) completed the second survey. The timing of the survey and social events in Japan related to the COVID‐19 pandemic are shown in Figure S1.

### Measures

#### Explanatory variables

This study's explanatory variables included participant demographics, length of school closure, diagnostic status of neurodevelopmental disorders, and parental depression. Regarding demographics, the Wave 1 survey measured participating youths' sex, age, school grade level, number of siblings, respondent's sex, age, nationality (Japanese; other), marital status (married [living together]; married [living separately]; single), and annual family income. Family income was assessed using an 11‐point Likert scale (1 = less than 1 million JPY; 2 = 1 million or more, but less than 2 million JPY; …; 10 = 9 million or more, but less than 10 million JPY; 11 = 10 million JPY or more). The Wave 2 survey measurements included parental educational level (secondary school; high school; vocational school; undergraduate; graduate; other), employment status (unemployed; self‐employed; part‐time; full‐time [not tenured]; full‐time [tenured]), rural‐urban status (rural; suburban; urban), and change in monthly family income between April 2019 and April 2020. Change in monthly family income was assessed using a 21‐point Likert scale (−10 = decreased more than 90%; −9 = decreased 81%−90%; …; −1 = decreased 1%−10%; 0 = not changed; 1 = increased 1%−10%; …; 9 = increased 81%−90%; 10 = increased more than 90%). All categorical variables were converted to binary variables (e.g., “suburban” response to the rural‐urban status item was integrated into the “urban” response) to make the data easier to interpret.

Participants provided the school closing and reopening dates, which were used to calculate the total length of school closure. Since school closure was a request, not an order, by the Japanese government, the total length of school closure varied, thus possibly explaining any variance in students' emotional/behavioral problems.

To assess a child's diagnostic status, parents provided “yes/no” answers to the question “Is your child diagnosed with the following disorders/disabilities?” for each neurodevelopmental disorder described in the DSM‐5 (American Psychiatric Association, 2013). Considering the possibility that some children could show complex symptoms or impairment and have not received a final diagnosis, respondents were asked to indicate neurodevelopmental disorders confirmed or suspected by their psychiatrist.

Parental depression was assessed using the 9‐item Patient Health Questionnaire (PHQ‐9; Kroenke et al., 2001; Kroenke, Spitzer, Williams, et al., 2010). Studies have found the Japanese version of PHQ‐9 to have good reliability and validity (Muramatsu et al., 2007). Cronbach's alpha in the current study was *α* = 0.891.

#### Response variables

Response variables (i.e., outcome variables), were youths' emotional/behavioral problems, as measured by the Strengths and Difficulties Questionnaire (SDQ; Goodman, 1997). This 25‐item questionnaire measures four domains of difficulties (emotional problems, conduct problems, hyperactivity/inattention, and peer relationship problems), one domain of strength (prosocial behavior), and the child's total difficulties (Warnick et al., 2008). Each subscale has norms to distinguish between normal, borderline, and clinical levels, according to the child's sex and age group. Borderline and clinical levels represent scores in the top 20% and 10%, respectively, of each subscale, except the prosocial behavior subscale, which defines borderline and clinical levels as scores in the bottom 20% and 10%, respectively. The SDQ has self‐, parent‐, and teacher‐report forms; the parent‐report form was used in the current study. The reliability and validity of the Japanese version of the SDQ have been confirmed by Moriwaki and Kamio (2014). Cronbach's alphas of emotional problems, conduct problems, hyperactivity/inattention, peer relationship problems, and prosocial behavior in the current study were *α* = 0.735, 0.627, 0.742, 0.551, 0.767, respectively.

### Statistical analysis

All data analyses were performed using IBM SPSS 25.0 and Mplus version 8.4. First, the descriptive statistics of all research variables were calculated. Factors associated with dropouts at Wave 2 were then explored by *t*‐tests for continuous variables and chi‐square tests for categorical variables. In the Japanese educational system, students enter the first grade in April, when they are six years old. Since Wave 1 was conducted in March, the number of 6‐year‐olds was small (*n* = 34; 0.71%), and inappropriate for the following analyses. Therefore, children's school grade levels, instead of their ages, were used in the analyses. Missing values were addressed by multiple imputations using Bayesian analysis (Rubin, 1987; Schafer, 1997), with a maximum iteration of 10,000, which finally generated 100 complete datasets. The multiple imputations used weighted values created according to the entire population at each prefecture, sex, and school grade level. The weighted values were also used in the following analyses.

Second, to examine the first hypothesis that children's emotional/behavioral problems increased during the COVID‐19 pandemic, the proportions of clinical‐level problems at Waves 1 and 2 were calculated and compared to those of a national Japanese sample (https://www.sdqinfo.org/norms/JapaneseNorms.html). Higher proportions of clinical‐level problems found at Wave 2 compared to Wave 1 or a national sample would be interpreted as an increase in emotional/behavioral problems in children and adolescents.

Third, logistic regression analyses were conducted to test the second hypothesis regarding the factors that could predict increases in children's emotional/behavioral problems during the COVID‐19 pandemic. The response variable was a binary variable indicating whether a child exhibited a clinical‐level problem at Wave 2, according to the cutoff points of the SDQ subscales. All explanatory variables, including SDQ scores at Wave 1, were entered simultaneously. The prefectures where participants lived were used as an auxiliary variable. The second hypothesis would be supported if the *p*‐value of the length of school closure was less than 0.05 and its odds ratio (QR) was more than 1.00.

## RESULTS

### Descriptive characteristics

Descriptive statistics for the research variables are shown in Tables 1 and 2. Most participants were Japanese (*n* = 4793%; 99.85%); therefore, nationality was used as an additional auxiliary variable, not an explanatory variable, in the following analyses. Monthly family income tended to be lower in April 2020 than in April 2019. The mean length of school closure at Wave 1 was less than 1 week.

**TABLE 1:** Descriptive statistics of the explanatory variables

	*n*	(%)	*M*	(SD)	Missing	(%)
Child's sex					0	(0.00)
Male	2400	(50.00)				
Female	2400	(50.00)				
Child's age (years)			12.41	(3.46)	0	(0.00)
Respondent's sex					0	(0.00)
Male	2633	(54.85)				
Female	2167	(45.15)				
Respondent's age (years)			44.19	(5.87)	0	(0.00)
Nationality					0	(0.00)
Japanese	4793	(99.85)				
Other	7	(0.15)				
Marital status					0	(0.00)
Married	4468	(93.08)				
Not married (single)	332	(6.92)				
Respondent's educational level					953	(19.85)
Undergraduate or more	1972	(51.26)				
Other	1875	(48.74)				
Respondent's employment status					953	(19.85)
Full‐time (tenured)	2420	(62.91)				
Other	1427	(37.09)				
Partner's educational level^a^					953	(19.85)
Undergraduate or more	1530	(42.24)				
Other	2092	(57.76)				
Partner's employment status^a^					953	(19.85)
Full‐time (tenured)	1854	(51.19)				
Other	1768	(48.81)				
Number of siblings			1.16	(0.93)	0	(0.00)
Rural‐urban status					953	(19.85)
Rural	687	(14.31)				
Suburban	1993	(41.52)				
Urban	1167	(24.31)				
Annual family income			7.33	(2.60)	0	(0.00)
Change in monthly income			‐1.44	(2.70)	953	(19.85)
Length of school closure (days)						
At Wave 1			5.4	(3.7)	1019	(21.2)
At Wave 2 (in total)			69.7	(15.5)	66	(1.7)
Total number of NDD diagnosis			0.15	(0.54)	25	(0.52)
0	4327	(90.6)				
1	295	(6.2)				
2 or more	153	(3.2)				

*Note. N* = 4800

Abbreviation: NDD, neurodevelopmental disorders

^a^ Participants who were not married (*n* = 225) were excluded.

**TABLE 2:** Descriptive statistics of parental depression and Children's emotional/behavioral problems in both waves

	Wave 1 (*N* = 4800)				Wave 2 (*N* = 3847)			
	*N*	(*%*)	*M*	(SD)	*n*	(*%*)	*M*	(SD)
Parental depression (PHQ‐9)			2.44	(4.01)			4.07	(5.05)
None‐minimal (4 or less)	3904	(81.5)			2542	(66.6)		
Mild (5 to 9)	561	(11.7)			743	(19.5)		
Moderate (10 to 14)	216	(4.5)			339	(8.9)		
Moderate to severe (15 to 19)	72	(1.5)			133	(3.5)		
Severe (20 or more)	35	(0.7)			59	(1.5)		
Emotional/behavioral problems (SDQ)								
Emotional problems			2.07	(2.19)			2.10	(2.23)
Normal	2979	(62.9)			2465	(65.4)		
Borderline	874	(18.4)			410	(10.9)		
Clinical	885	(18.7)			894	(23.7)		
Conduct problems			2.19	(1.86)			2.29	(1.89)
Normal	3375	(71.2)			2417	(64.1)		
Borderline	548	(11.6)			525	(13.9)		
Clinical	815	(17.2)			827	(21.9)		
Hyperactivity/Inattention			3.56	(2.37)			3.78	(2.33)
Normal	3076	(64.9)			1905	(50.5)		
Borderline	669	(14.1)			560	(14.9)		
Clinical	993	(21.0)			1304	(34.6)		
Peer relationship problems			2.61	(1.90)			2.88	(1.90)
Normal	2511	(53.0)			1754	(46.5)		
Borderline	849	(17.9)			706	(18.7)		
Clinical	1378	(29.1)			1309	(34.7)		
Prosocial behavior			5.40	(2.42)			5.15	(2.38)
Normal	3182	(67.2)			2349	(62.3)		
Borderline	667	(14.1)			559	(14.8)		
Clinical	889	(18.8)			861	(22.8)		
Total difficulties			10.44	(6.09)			11.04	(6.26)
Normal	2886	(60.9)			1819	(48.3)		
Borderline	723	(15.3)			819	(21.7)		
Clinical	1129	(23.8)			1131	(30.0)		

Abbreviations: PHQ‐9, 9‐item Patient Health Questionnaire; SDQ, Strengths and Difficulties Questionnaire

Comparative analyses of participants who did (completers) or did not (noncompleters) complete the Wave 2 survey indicated that completers were more likely to have older children (*t* = −4.46, *df* = 4798, *p* < .001), be male (*χ* ^2^ = 90.34, *df* = 1, *p* < .001), be married (*χ* ^2^ = 4.63, *df* = 1, *p* = .031), have fewer children (*t* = 2.67, *df* = 4798, *p* = .008), and have a higher income (*t* = −3.44, *df* = 4798, *p* < .001). For the SDQ, standardized mean differences and 95% confidence intervals (CIs) between completers and non‐completers for the peer relationship problems (0.17 [0.04−0.31]) and prosocial behavior (−0.23 [−0.05−−0.40]) suggested that completers' children tended to show more peer relationship problems and less prosocial behavior. There were no statistically significant differences for any other variables. Detailed descriptions of the differences between completers and noncompleters are shown in Table S2.

### Increases in Children's emotional/behavioral problems

As shown in Table 2, from Waves 1 to 2 there were increases in the absolute number of participants who reported clinical‐level emotional symptoms, conduct problems, hyperactivity/inattention, and severe parental depression, while the total number of participants decreased due to attrition. The highest proportions of clinical‐level problems at Wave 2 were observed for neurodevelopmental disorders (emotional symptoms = 43.6% [39.0−48.2]; conduct problems = 35.8% [31.3−40.3]; hyperactivity/inattention = 58.4% [53.8−63.0]; peer relationship problems = 51.8% [47.1−56.5]) and annual income of less than 2 million JPY (lack of prosocial behavior = 33.0% [25.8−40.2]).

**FIGURE 1:**
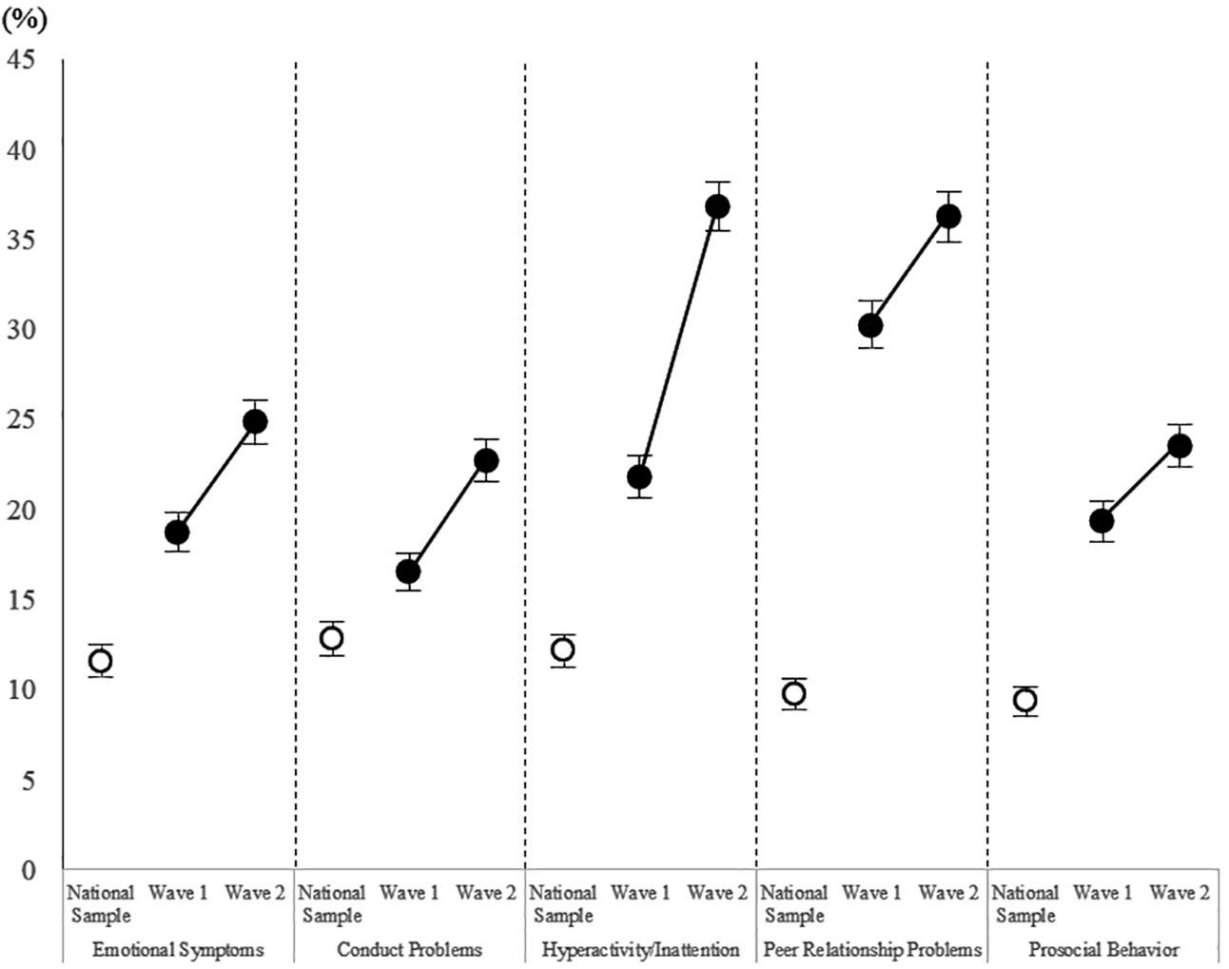
Increases in the proportions of clinical‐level problems measured by the Parent‐Report SDQ Note: Error bar means 95% confidence interval

As illustrated in the Figure 1, the estimates and 95% CIs of the proportions of clinical‐level problems were higher at Wave 2 than at Wave 1 in all five SDQ subscales: emotional symptoms (18.7% [17.6−19.8] at Wave 1; 24.8% [23.6−26.0] at Wave 2), conduct problems (16.5% [15.4−17.6] at Wave 1; 22.7% [21.5−23.9] at Wave 2), hyperactivity/inattention (21.8% [20.6−23.0] at Wave 1; 36.8% [35.4−38.2] at Wave 2), peer relationship problems (30.2% [28.9−31.5] at Wave 1; 36.2% [34.8−37.6] at Wave 2), and lack of prosocial behavior (19.3% [18.2−20.4] at Wave 1; 23.5% [22.3−24.7] at Wave 2). It is also worth noting that all five outcomes at Wave 1 were still higher than the national sample. Detailed descriptions of the estimated proportions and 95% CIs for clinical‐level problems are displayed in Table S3.

### Risk factors for the increase in Children's emotional/behavioral problems

Logistic regression analysis revealed that children's school grade levels predicted proportions of clinical‐level emotional symptoms (OR = 0.94 [0.91−0.98], *p* = .005) and hyperactivity/inattention at Wave 2 (OR = 0.91 [0.87−0.94], *p* < .001). Additionally, lower annual family income predicted children's classification at the clinical level for lack of prosocial behavior at Wave 2 (OR = 0.95 [0.90−0.99], *p* = .028). More hyperactivity/inattention at Wave 1 predicted a higher proportion of clinical‐level conduct problems at Wave 2 (OR = 1.10 [1.03−1.16], *p* = .002). Parental depression at Wave 2 was associated with all four problem subscales of the SDQ at Wave 2: emotional problems (OR = 1.11 [1.19−1.14], *p* < .001), conduct problems (OR = 1.10 [1.07−1.13], *p* < .001), hyperactivity/inattention (OR = 1.07 [1.05−1.10], *p* < .001), and peer relationship problems (OR = 1.07 [1.04−1.09], *p* < .001). No other explanatory variables, including total length of school closure, predicted children's emotional/behavioral problems at Wave 2. The summary of the results of this analysis are presented in Table S4.

Further descriptive analysis revealed that an increased proportion of clinical‐level hyperactivity/inattention was observed for children in grades 1−7 (Figure S2). Similarly, an increased proportion of clinical‐level emotional symptoms was observed for children in grades 1−3 (19.6% [17.4−21.8] at Wave 1; 31.7% [29.1−34.3] at Wave 2; Table S3). Regarding children's prosocial behavior, the highest proportion of clinical‐level problems at Wave 2 (33.0% [25.8−40.2]) was observed in children with an annual family income of less than 2 million JPY (approximately 18.8 thousand US Dollars, 13.5 thousand British Pounds, or 15.5 thousand euros; Table S3).

## DISCUSSION

This study aimed to investigate increases in emotional/behavioral problems and related risk factors among children and adolescents during the COVID‐19 pandemic. Results indicated significant increases in proportions of clinical‐level emotional/behavioral problems. Additional analysis revealed increased emotional symptoms and hyperactivity/inattention among children in grades 1–3, and decreased prosocial behavior in those with a low family income. Length of school closure, however, did not predict emotional/behavioral problems. More research is needed to explore why children may exhibit more behavioral problems during a nationwide severe epidemic and how this could be prevented.

In line with the first hypothesis, the proportion of clinical‐level emotional/behavioral problems in the current sample was higher at Wave 2 compared to Wave 1 and another Japanese national sample that completed the SDQ (Moriwaki & Kamio, 2014). Some cross‐sectional studies have reported high levels of internalizing problems, such as depression or anxiety, in children during the COVID‐19 pandemic (Xie et al., 2020; Zhou et al., 2020). The results of the current prospective cohort study supported these previous findings and extended them to externalizing problems.

Regarding risk factors, increases in youths' clinical‐level emotional/behavioral problems were predicted by two demographic factors: lower school grade level and lower annual family income. This result was in line with several studies suggesting the impact of lower income on youths' psychosocial problems (Langley et al., 2007; Thapar et al., 2012). The current findings identified vulnerable groups that should be the target of additional care for preventing mental health problems during the COVID‐19 pandemic.

Although a neurodevelopmental disorder diagnosis was not a significant predictor of increases in emotional/behavioral problems, this does not mean that children with neurodevelopmental disorders were not affected by the pandemic. Instead, they were affected in a similar way as those without neurodevelopmental disorders. The cumulative effect of neurodevelopmental disorders and the COVID‐19 pandemic placed children at higher risk for clinical‐level emotional/behavioral problems (McGinty et al., 2020).

Notably, contrary to the second hypothesis, school closure itself was not a significant predictor of emotional/behavioral problems. Since other researchers have proposed several possible risk factors that may co‐occur with school closure (Lancet Child Adolescent Health, 2020; Viner et al., 2020), such as child abuse (Galea et al., 2020), social deprivations (Orben et al., 2020), or unfavorable changes in lifestyle behaviors (Pietrobelli et al., 2020), the effects of these factors on emotional/behavioral problems should be examined in future research.

### Limitations

Despite our study's informative findings and strengths, the results should be interpreted in light of the following limitations. First, 953 (19.9%) withdrawals occurred between Waves 1 and 2, implying that the current results could be influenced by possible selection bias. For example, non‐completers tended to have a lower family income and showed more prosocial behavior. This indicates that attrition in the study could be the common effect (Hernán et al., 2004) of annual income and prosocial behavior. While this study statistically addressed the effect of sample attrition with the multiple imputation method, future research will need to take substantial measures to prevent sample dropout.

Second, all measurements in this study relied on parent reports; thus, the influence of measurement and common method biases (Podsakoff et al., 2003) should be considered. Although this study addressed the common method bias of negative affectivity by measuring and controlling for parental depression, a single informant would still not be enough to fully capture the nature of children and adolescents' mental health problems. Measurement bias may have also occurred in the self‐report section for the length of school closure. Accordingly, clinicians' ratings of neurodevelopmental disorders, children's self‐reports on depression/anxiety, the official record of school closure, or any other valid measurements should be considered in future research.

Third, this study investigated increases in emotional/behavioral problems over two months; thus, the long‐term effects of the pandemic and school closures remain unclear. Understanding the long‐term picture of pandemic‐related psychopathology in children and adolescents will help identify vulnerable individuals and facilitate the development of early interventions. Follow‐up studies that describe the longitudinal effects of the COVID‐19 pandemic on children's emotional/behavioral problems will be needed.

## CONCLUSION

Even with the above limitations, this study provides valuable insight on the emotional/behavioral problems in children and adolescents during the COVID‐19 pandemic. Increases in proportions of clinical‐level problems were observed in all emotional/behavioral domains: emotional symptoms, conduct problems, hyperactivity/inattention, peer relationship problems, and a lack of prosocial behavior. Appropriate prevention and early intervention programs should be provided, especially for children in grades 1−3, or those with a low family income or neurodevelopmental disorders.

## CONFLICT OF INTEREST

No conflicts declared.

## ETHICS APPROVAL

Shinshu University Ethics Committee on Educational Research approved this study to be conducted.

## AUTHOR CONTRIBUTIONS

The first author conceptualized and designed the study, did the literature review, cleaned and analyzed the data, and wrote the first draft of the manuscript. The second author interpreted data and reviewed and revised the manuscript. All authors approved the final manuscript for submission and agreed to be accountable for all aspects of the work.

## PATIENT CONSENT STATEMENT

All study participants provided informed consent to participate in this survey.

## Supporting information

Supporting Information1

## Data Availability

Data used in this study are available upon request.
