# Supplementary material for: Prevalence of clinical‐level emotional/behavioral problems in schoolchildren during the coronavirus disease 2019 pandemic in Japan: A prospective cohort study
Source: JCPP Adv. 2021 Apr 28;1(1):e12007. doi: 10.1111/jcv2.12007 (PMC8206658; doi:10.1111/jcv2.12007)
Supplement: Supplementary file 1 — Supporting Information1 [file JCV2-1-e12007-s001.docx]

**Table S1.** Summary of Literature Regarding Mental Health Issues in Children and Adolescents During Infectious Disease Epidemics.

| **Author** | **Year** | **Country** | **Research design** | **Sample** | **Child’s age (Years)** | **N** | **Measure** | **Mental health outcome** | **Summary of results** |
| --- | --- | --- | --- | --- | --- | --- | --- | --- | --- |
| Koller et al. | 2006 | Canada | Ethnographic interview | Pediatric patients affected by SARS, their parents, and their pediatric health care providers | 6 to 18 | 5 | Interview and health care records | Emotional impact of SARS | Hospitalized children often experienced sadness. |
| Zhang et al. | 2020 | China | Cross-sectional | Parents of children with ADHD diagnosis | 6 to 15 (Mean = 9.43) | 241 | Parent-report | Behavioral and emotional symptoms | Parents reported that their child's ADHD symptoms "worsened" compared to their normal state. |
| Xie et al. | 2020 | China | Cross-sectional | Community cohort during the COVID-19 outbreak | 2nd to 6th grade | 1,784 | Self-report | Depression, anxiety, and worry | The proportions of children who reported depressive or anxiety symptoms during the COVID-19 outbreak were 22.6% and 18.9%, respectively (severity not available). |
| Zhou, Zhang, et al. | 2020 | China | Cross-sectional | Community cohort during the COVID-19 outbreak | 12 to 18 (Median = 16) | 8,079 | Self-report | Depression and anxiety symptoms | The proportions of adolescents who reported severe depression and anxiety during the COVID-19 outbreak were 2.7% and 3.0%, respectively. |
| Colizzi et al. | 2020 | Italy | Cross-sectional | Children and adolescents with ASD diagnosis | Mean = 13.0 | 527 | Parent-report | Behavioral problems | The proportions of parents who reported the intensity and frequency of their child's behavioral problems worsened were 35.5% and 41.5%, respectively. |
| Bobo et al. | 2020 | France | Cross-sectional | Children and adolescents with ADHD diagnosis | Mean = 10.5 | 538 | Parent-report | Well-being | The proportions of parents who reported their child's well-being worsened or improved were 34.7% and 31.0%, respectively. |
| Fish et al. | 2020 | United States | Cross-sectional | Youth in chat-based support group for LGBTQ youth | Anonymous | 159 | Chat transcripts | Experienced challenges and resources | Youth experienced several kinds of intrapersonal, interpersonal, and structural challenges. They also found online support and resources to be useful. |
| Saurabh & Ranjan | 2020 | India | Cross-sectional | Children at home/facility quarantine | 9 to 18 (Mean = 15.4) | 121 | Interview | Psychological symptoms | Quarantined children and adolescents, compared to non-quarantined ones, were more likely to report fear (61.98%), nervousness (60.33%), annoyance (57.85%), and loneliness (48.76%). |
| Qi, Liu, et al. | 2020 | China | Cross-sectional | Online sample (snowball sampling) | 11 to 20 | 9,554 | Self-report | Anxiety | The proportion of children who reported severe anxiety symptoms during the COVID-19 pandemic was 1.5%. |
| Islam et al. | 2020 | Bangladesh | Cross-sectional | Online sample | 13 to 20 | 306 | Self-report | Anxiety | The proportion of children who reported moderate anxiety symptoms during the COVID-19 pandemic was 31.0%. |
| Asbury et al. | 2020 | United Kingdom | Cross-sectional | Parents from existing research networks, special schools, and online platforms | 5 to 18 (Mean = 9) | 241 | Parent-report (free description) | Behavioral and emotional symptoms | The three most frequently reported problems in children were change in routine (n = 84; 34.9%), anxiety (n = 60; 24.9%), and challenging behavior (n = 44; 18.3%). |
| Zhou, Yuan, et al. | 2020 | China | Cross-sectional | Online female sample (snowball sampling) | 11 to 18 (Median = 15) | 4,805 | Self-report | Depression | The proportion of female adolescents who reported depressive symptoms during the COVID-19 pandemic was 39.5%. |
| Dumas et al. | 2020 | Canada | Cross-sectional | Online sample and pre-existing participants from a previous study | 14 to 18 (Mean = 16.68) | 1,054 | Self-report | Substance use | There were decreases in the percentages of most substance use problems: binge drinking (15.7 to 9.8%), cannabis use (17.0 to 13.8%), and vaping (16.6 to 11.5%). |
| Qi, Zhou, et al. | 2020 | China | Cross-sectional | Adolescents from social networking service groups | 14 to 18  (Median = 16) | 7,202 | Self-report | Depression and anxiety | The proportions of children who reported mild to severe depressive or anxiety symptoms during the COVID-19 pandemic were 44.5% and 38.0%, respectively. |
| Duan et al. | 2020 | China | Cross-sectional | Convenience sampling | 7 to 18 | 3,613 | Self-report | Depression, anxiety, and Internet addiction | The proportions of children who reported clinical depressive symptoms or internet addiction during the COVID-19 pandemic were 22.3% and 6.0%, respectively. |

*Note:* Databases: PsycInfo and Medline. Period of publication: before August 14, 2020. Restrictions on language: None. Search terms: “covid-19 or coronavirus or 2019-ncov or sars-cov-2 or cov-19 or sars or mers” AND “children or adolescents or youth or child or teenager” AND “mental health or mental illness or mental disorder or problem behavior or behavior problem.” These searches obtained 635 results, including 250 articles unrelated to epidemics, 258 related to infectious disease but not in children, and 18 related to infectious disease and children but not focused on mental health. The remaining 109 articles were primarily focused on children’s mental health or behavioral problems during an epidemic. Of these 109 articles, 82 were commentaries, letters, editorials, literature reviews, or otherwise did not present original empirical data. Extracting 12 papers without descriptive analyses of the intensity or frequency of emotional/behavioral problems, authors identified the above 15 studies demonstrating children’s emotional/behavioral problems during the SARS or COVID-19 epidemics.

Asbury, K., Fox, L., Deniz, E., Code, A., & Toseeb, U. (2020). How is covid-19 affecting the mental health of children with special educational needs and disabilities and their families? *Journal of Autism and Developmental Disorders*. https://doi.org/10.1007/s10803-020-04577-2

Bobo, E., Lin, L., Acquaviva, E., Caci, H., Franc, N., Gamon, L., Picot, M. C., Pupier, F., Speranza, M., Falissard, B., & Purper-Ouakil, D. (2020). Comment les enfants et adolescents avec le trouble déficit d’attention/hyperactivité (TDAH) vivent-ils le confinement durant la pandémie COVID-19? [How do children and adolescents with Attention Deficit Hyperactivity Disorder (ADHD) experience lockdown during the COVID-19 outbreak?] *L'Encéphale [Encephale], 46*(3, Supplement), S85-S92. https://doi.org/10.1016/j.encep.2020.05.011

Colizzi, M., Sironi, E., Antonini, F., Ciceri, M. L., Bovo, C., & Zoccante, L. (2020). Psychosocial and behavioral impact of COVID-19 in autism spectrum disorder: An online parent survey. *Brain Sciences, 10*(6), 341. Retrieved from https://www.mdpi.com/2076-3425/10/6/341

Duan, L., Shao, X., Wang, Y., Huang, Y., Miao, J., Yang, X., & Zhu, G. (2020). An investigation of mental health status of children and adolescents in china during the outbreak of COVID-19. *Journal of Affective Disorders, 275*, 112-118. https://doi.org/10.1016/j.jad.2020.06.029

Dumas, T. M., Ellis, W., & Litt, D. M. (2020). What does adolescent substance use look like during the COVID-19 pandemic? Examining changes in frequency, social contexts, and pandemic-related predictors. *Journal of Adolescent Health*. https://doi.org/10.1016/j.jadohealth.2020.06.018

Fish, J. N., McInroy, L. B., Paceley, M. S., Williams, N. D., Henderson, S., Levine, D. S., & Edsall, R. N. (2020). “I'm kinda stuck at home with unsupportive parents right now”: LGBTQ youths' experiences with COVID-19 and the importance of online support. *Journal of Adolescent Health.* https://doi.org/10.1016/j.jadohealth.2020.06.002

Islam, M. S., Ferdous, M. Z., & Potenza, M. N. (2020). Panic and generalized anxiety during the COVID-19 pandemic among Bangladeshi people: An online pilot survey early in the outbreak. *Journal of Affective Disorders, 276*, 30-37. https://doi.org/10.1016/j.jad.2020.06.049

Koller, D. F., Nicholas, D. B., Goldie, R. S., Gearing, R., & Selkirk, E. K. (2006). When family-centered care is challenged by infectious disease: Pediatric health care delivery during the SARS outbreaks. *Qualitative Health Research, 16*(1), 47-60. https://doi.org/10.1177/1049732305284010

Qi, H., Liu, R., Chen, X., Yuan, X.-F., Li, Y.-Q., Huang, H.-H., Zheng, Y., &Wang, G. (2020). Prevalence of anxiety and associated factors for Chinese adolescents during the COVID-19 outbreak. *Psychiatry and Clinical Neurosciences*. https://doi.org/10.1111/pcn.13102

Qi, M., Zhou, S.-J., Guo, Z.-C., Zhang, L.-G., Min, H.-J., Li, X.-M., & Chen, J.-X. (2020). The effect of social support on mental health in Chinese adolescents during the outbreak of COVID-19. *The Journal of Adolescent Health, 67*(4), 514-518. https://doi.org/10.1016/j.jadohealth.2020.07.001

Saurabh, K., & Ranjan, S. (2020). Compliance and psychological impact of quarantine in children and adolescents due to Covid-19 pandemic. *Indian Journal of Pediatrics, 87*(7), 532-536. https://doi.org/10.1007/s12098-020-03347-3

Xie, X., Xue, Q., Zhou, Y., Zhu, K., Liu, Q., Zhang, J., & Song, R. (2020). Mental health status among children in home confinement during the coronavirus disease 2019 outbreak in Hubei province, China. *JAMA Pediatrics, 174*(9), 898-900. https://doi.org/10.1001/jamapediatrics.2020.1619

Zhang, J., Shuai, L., Yu, H., Wang, Z., Qiu, M., Lu, L., Cao, X., Xia, W., Wang, Y., & Chen, R. (2020). Acute stress, behavioural symptoms and mood states among school-age children with attention-deficit/hyperactive disorder during the COVID-19 outbreak. *Asian Journal of Psychiatry, 51*, 102077. https://doi.org/10.1016/j.ajp.2020.102077

Zhou, J., Yuan, X., Qi, H., Liu, R., Li, Y., Huang, H., Chen, X., & Wang, G. (2020). Prevalence of depression and its correlative factors among female adolescents in China during the coronavirus disease 2019 outbreak. *Globalization and Health, 16*(1), 69. https://doi.org/10.1186/s12992-020-00601-3

Zhou, S.-J., Zhang, L.-G., Wang, L.-L., Guo, Z.-C., Wang, J.-Q., Chen, J.-C., Liu, M., Chen, X., & Chen, J.-X. (2020). Prevalence and socio-demographic correlates of psychological health problems in Chinese adolescents during the outbreak of covid-19. *European Child & Adolescent Psychiatry, 29*, 749-758. https://doi.org/10.1007/s00787-020-01541-4

**Figure S1.** Cumulative Number of COVID-19 Cases, Social Events in Japan, and Research Events.


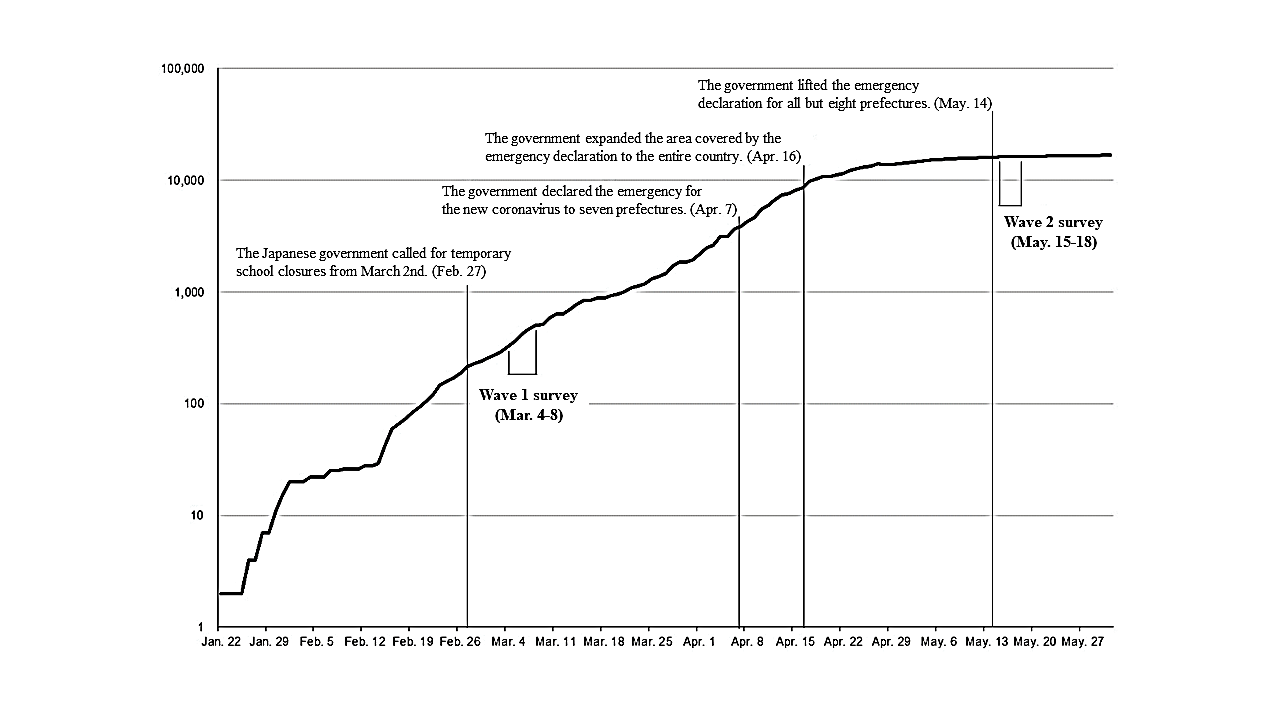


**Table S2.** Comparisons of Wave 1 Participants Who Did or Did Not Complete Wave 2.

|  | Completer (*N* = 3,847) | | | | Non-completer (*N* = 953) | | | | *χ^2^* | *p* | *t* | *p* |
| --- | --- | --- | --- | --- | --- | --- | --- | --- | --- | --- | --- | --- |
|  | ***n*** | ***(%)*** | ***M*** | ***(SD)*** | ***n*** | ***(%)*** | ***M*** | ***(SD)*** |  |  |  |  |
| Child's sex |  |  |  |  |  |  |  |  | 0.30 | .587 |  |  |
| Male | 1931 | (50.2) |  |  | 469 | (49.2) |  |  |  |  |  |  |
| Female | 1916 | (49.8) |  |  | 484 | (50.8) |  |  |  |  |  |  |
| Child's age |  |  | 12.52 | (3.45) |  |  | 11.97 | (3.46) |  |  | -4.46 | < .001 |
| Respondent's sex |  |  |  |  |  |  |  |  | 90.40 | < .001 |  |  |
| Male | 2241 | (58.3) |  |  | 392 | (41.1) |  |  |  |  |  |  |
| Female | 1606 | (41.7) |  |  | 561 | (58.9) |  |  |  |  |  |  |
| Respondent's age |  |  | 44.58 | (5.82) |  |  | 42.63 | (5.80) |  |  | -9.24 | < .001 |
| Respondent's nationality |  |  |  |  |  |  |  |  | 0.34 | .563 |  |  |
| Japanese | 3842 | (99.9) |  |  | 951 | (99.8) |  |  |  |  |  |  |
| Other | 5 | (0.1) |  |  | 2 | (0.2) |  |  |  |  |  |  |
| Respondent's marital status |  |  |  |  |  |  |  |  | 4.63 | .031 |  |  |
| Married | 3596 | (93.5) |  |  | 872 | (91.5) |  |  |  |  |  |  |
| Single | 251 | (6.5) |  |  | 81 | (8.5) |  |  |  |  |  |  |
| Number of siblings |  |  | 1.14 | (0.93) |  |  | 1.23 | (0.94) |  |  | 2.67 | .008 |
| Annual income |  |  | 7.40 | (2.60) |  |  | 7.08 | (2.58) | 13.65 | .003 | -3.44 | < .001 |
| Very low (less than 2 mil. JPY) | 137 | (3.6) |  |  | 41 | (4.3) |  |  |  |  |  |  |
| Low (2 to 4 mil. JPY) | 381 | (9.9) |  |  | 116 | (12.2) |  |  |  |  |  |  |
| Middle (4 to 8 mil. JPY) | 1941 | (50.5) |  |  | 508 | (53.3) |  |  |  |  |  |  |
| High (more than 8 mil. JPY) | 1388 | (36.1) |  |  | 288 | (30.2) |  |  |  |  |  |  |
| NDD diagnosis |  |  |  |  |  |  |  |  |  |  |  |  |
| Intellectual Disability | 79 | (2.1) |  |  | 10 | (1.0) |  |  |  |  |  |  |
| Global Developmental Delay | 44 | (1.1) |  |  | 4 | (0.4) |  |  |  |  |  |  |
| Language Disorder | 18 | (0.5) |  |  | 3 | (0.3) |  |  |  |  |  |  |
| Speech Sound Disorder | 4 | (0.1) |  |  | 1 | (0.1) |  |  |  |  |  |  |
| Childhood-Onset Fluency Disorder (Stuttering) | 25 | (0.6) |  |  | 9 | (0.9) |  |  |  |  |  |  |
| Social (Pragmatic) Communication Disorder | 37 | (1.0) |  |  | 4 | (0.4) |  |  |  |  |  |  |
| Autism Spectrum Disorder | 147 | (3.8) |  |  | 30 | (3.1) |  |  |  |  |  |  |
| Attention-Deficit/Hyperactivity Disorder | 125 | (3.2) |  |  | 26 | (2.7) |  |  |  |  |  |  |
| Specific Learning Disorder | 9 | (0.2) |  |  | 1 | (0.1) |  |  |  |  |  |  |
| Developmental Coordination Disorder | 16 | (0.4) |  |  | 6 | (0.6) |  |  |  |  |  |  |
| Stereotypic Movement Disorder | 3 | (0.1) |  |  | 2 | (0.2) |  |  |  |  |  |  |
| Tic Disorders | 76 | (2.0) |  |  | 18 | (1.9) |  |  |  |  |  |  |
| Other psychiatric disorders | 19 | (0.5) |  |  | 7 | (0.7) |  |  |  |  |  |  |
| Total number of NDD diagnoses |  |  | 0.15 | (0.56) |  |  | 0.12 | (0.46) | 2.28 | .320 | -1.65 | .100 |
| 0 | 3454 | (90.3) |  |  | 873 | (91.9) |  |  |  |  |  |  |
| 1 | 244 | (6.4) |  |  | 51 | (5.4) |  |  |  |  |  |  |
| 2 or more | 127 | (3.3) |  |  | 26 | (2.7) |  |  |  |  |  |  |
| Parental depression (PHQ-9) |  |  | 2.40 | (3.97) |  |  | 2.58 | (4.18) | 1.38 | .848 | 1.26 | .207 |
| None-minimal (4 or less) | 3136 | (81.7) |  |  | 768 | (80.8) |  |  |  |  |  |  |
| Mild (5 to 9) | 449 | (11.7) |  |  | 112 | (11.8) |  |  |  |  |  |  |
| Moderate (10 to 14) | 170 | (4.4) |  |  | 46 | (4.8) |  |  |  |  |  |  |
| Moderate to severe (15 to 19) | 56 | (1.5) |  |  | 16 | (1.7) |  |  |  |  |  |  |
| Severe (20 or more) | 26 | (0.7) |  |  | 9 | (0.9) |  |  |  |  |  |  |
| Emotional/behavioral problems (SDQ) |  |  |  |  |  |  |  |  |  |  |  |  |
| Emotional problems |  |  | 2.07 | (2.20) |  |  | 2.11 | (2.14) | 1.13 | .569 | 0.53 | .593 |
| Normal | 2393 | (63.1) |  |  | 586 | (61.9) |  |  |  |  |  |  |
| Borderline | 688 | (18.1) |  |  | 186 | (19.6) |  |  |  |  |  |  |
| Clinical | 710 | (18.7) |  |  | 175 | (18.5) |  |  |  |  |  |  |
| Conduct problems |  |  | 2.19 | (1.86) |  |  | 2.20 | (1.84) | 1.78 | .412 | 0.12 | .905 |
| Normal | 2700 | (71.2) |  |  | 675 | (71.3) |  |  |  |  |  |  |
| Borderline | 429 | (11.3) |  |  | 119 | (12.6) |  |  |  |  |  |  |
| Clinical | 662 | (17.5) |  |  | 153 | (16.2) |  |  |  |  |  |  |
| Hyperactivity/Inattention |  |  | 3.56 | (2.36) |  |  | 3.55 | (2.41) | 2.19 | .334 | -0.11 | .909 |
| Normal | 2446 | (64.5) |  |  | 630 | (66.5) |  |  |  |  |  |  |
| Borderline | 534 | (14.1) |  |  | 135 | (14.3) |  |  |  |  |  |  |
| Clinical | 811 | (21.4) |  |  | 182 | (19.2) |  |  |  |  |  |  |
| Peer relationship problems |  |  | 2.65 | (1.91) |  |  | 2.48 | (1.87) | 4.28 | .118 | -2.48 | .013 |
| Normal | 1984 | (52.3) |  |  | 527 | (55.6) |  |  |  |  |  |  |
| Borderline | 680 | (17.9) |  |  | 169 | (17.8) |  |  |  |  |  |  |
| Clinical | 1127 | (29.7) |  |  | 251 | (26.5) |  |  |  |  |  |  |
| Prosocial behavior |  |  | 5.36 | (2.43) |  |  | 5.59 | (2.40) | 1.51 | .471 | 2.58 | .010 |
| Normal | 2531 | (66.8) |  |  | 651 | (68.7) |  |  |  |  |  |  |
| Borderline | 537 | (14.2) |  |  | 130 | (13.7) |  |  |  |  |  |  |
| Clinical | 723 | (19.1) |  |  | 166 | (17.5) |  |  |  |  |  |  |
| Total difficulties |  |  | 10.46 | (6.15) |  |  | 10.33 | (5.85) | 6.42 | .040 | -0.59 | .556 |
| Normal | 2305 | (60.8) |  |  | 581 | (61.4) |  |  |  |  |  |  |
| Borderline | 559 | (14.7) |  |  | 164 | (17.3) |  |  |  |  |  |  |
| Clinical | 927 | (24.5) |  |  | 202 | (21.3) |  |  |  |  |  |  |

*Note:* NDD = Neurodevelopmental Disorders; PHQ-9 = 9-item Patient Health Questionnaire; SDQ = Strengths and Difficulties Questionnaire.

**Table S3.** Proportions of Clinical-Level Emotional/Behavioral Problems Measured With Parent-Report SDQ.

|  |  |  | Emotional Symptoms | | Conduct Problems | | Hyperactivity/Inattention | | Peer Relationship Problems | | Prosocial Behavior | |
| --- | --- | --- | --- | --- | --- | --- | --- | --- | --- | --- | --- | --- |
|  |  |  | Wave 1 | Wave 2 | Wave 1 | Wave 2 | Wave 1 | Wave 2 | Wave 1 | Wave 2 | Wave 1 | Wave 2 |
|  |  | *n* | [95% CI] | [95% CI] | [95% CI] | [95% CI] | [95% CI] | [95% CI] | [95% CI] | [95% CI] | [95% CI] | [95% CI] |
| Overall |  | 4,800 | 18.7 | 24.8 | 16.5 | 22.7 | 21.8 | 36.8 | 30.2 | 36.2 | 19.3 | 23.5 |
|  |  |  | [17.6-19.8] | [23.6-26.0] | [15.4-17.6] | [21.5-23.9] | [20.6-23.0] | [35.4-38.2] | [28.9-31.5] | [34.8-37.6] | [18.2-20.4] | [22.3-24.7] |
| Child's sex | Male | 2,540 | 20.1 | 24.8 | 16.4 | 23.6 | 19.7 | 40.0 | 33.5 | 39.6 | 19.5 | 25.5 |
|  |  |  | [18.5-21.7] | [23.1-26.5] | [15.0-17.8] | [21.9-25.3] | [18.2-21.2] | [38.1-41.9] | [31.7-35.3] | [37.7-41.5] | [18.0-21.0] | [23.8-27.2] |
|  | Female | 2,260 | 17.2 | 23.3 | 16.9 | 20.9 | 24.1 | 32.4 | 26.8 | 31.9 | 19.3 | 19.8 |
|  |  |  | [15.6-18.8] | [21.6-25.0] | [15.4-18.4] | [19.2-22.6] | [22.3-25.9] | [30.5-34.3] | [25.0-28.6] | [30.0-33.8] | [17.7-20.9] | [18.2-21.4] |
| Child's school grade | 1 to 3 | 1,211 | 19.6 | 31.7 | 12.7 | 25.8 | 22.0 | 51.3 | 29.5 | 34.2 | 23.8 | 20.6 |
|  |  |  | [17.4-21.8] | [29.1-34.3] | [10.8-14.6] | [23.3-28.3] | [19.7-24.3] | [48.5-54.1] | [26.9-32.1] | [31.5-36.9] | [21.4-26.2] | [18.3-22.9] |
|  | 4 to 6 | 1,152 | 21.3 | 23.5 | 15.2 | 23.3 | 21.3 | 36.9 | 28.7 | 37.6 | 18.6 | 21.0 |
|  |  |  | [18.9-23.7] | [21.1-25.9] | [13.1-17.3] | [20.9-25.7] | [18.9-23.7] | [34.1-39.7] | [26.1-31.3] | [34.8-40.4] | [16.4-20.8] | [18.6-23.4] |
|  | 7 to 9 | 1,148 | 20.3 | 23.0 | 20.6 | 22.3 | 24.8 | 33.1 | 32.0 | 34.9 | 15.8 | 23.1 |
|  |  |  | [18.0-22.6] | [20.6-25.4] | [18.3-22.9] | [19.9-24.7] | [22.3-27.3] | [30.4-35.8] | [29.3-34.7] | [32.1-37.7] | [13.7-17.9] | [20.7-25.5] |
|  | 10 to 12 | 1,289 | 14.3 | 18.4 | 18.1 | 18.1 | 19.3 | 25.0 | 31.2 | 37.1 | 19.4 | 26.5 |
|  |  |  | [12.4-16.2] | [16.3-20.5] | [16.0-20.2] | [16.0-20.2] | [17.1-21.5] | [22.6-27.4] | [28.7-33.7] | [34.5-39.7] | [17.2-21.6] | [24.1-28.9] |
| Respondent's sex | Male | 2,695 | 19.2 | 24.8 | 18.1 | 24.2 | 24.3 | 39.4 | 31.6 | 39.1 | 19.5 | 23.5 |
|  |  |  | [17.7-20.7] | [23.2-26.4] | [16.6-19.6] | [22.6-25.8] | [22.7-25.9] | [37.6-41.2] | [29.8-33.4] | [37.3-40.9] | [18.0-21.0] | [21.9-25.1] |
|  | Female | 2,105 | 18.2 | 23.2 | 14.8 | 20.0 | 18.5 | 32.7 | 28.7 | 32.0 | 19.3 | 22.1 |
|  |  |  | [16.6-19.8] | [21.4-25.0] | [13.3-16.3] | [18.3-21.7] | [16.8-20.2] | [30.7-34.7] | [26.8-30.6] | [30.0-34.0] | [17.6-21.0] | [20.3-23.9] |
| Respondent's age | Less than 40 | 939 | 23.1 | 30.2 | 20.3 | 27.9 | 24.1 | 45.0 | 30.4 | 32.4 | 21.4 | 20.2 |
|  |  |  | [20.4-25.8] | [27.3-33.1] | [17.7-22.9] | [25.0-30.8] | [21.4-26.8] | [41.8-48.2] | [27.5-33.3] | [29.4-35.4] | [18.8-24.0] | [17.6-22.8] |
|  | 40 or more | 3,861 | 17.7 | 22.6 | 15.8 | 21.0 | 21.2 | 34.4 | 30.3 | 36.8 | 18.9 | 23.5 |
|  |  |  | [16.5-18.9] | [21.3-23.9] | [14.6-17.0] | [19.7-22.3] | [19.9-22.5] | [32.9-35.9] | [28.9-31.7] | [35.3-38.3] | [17.7-20.1] | [22.2-24.8] |
| Respondent's marital status | Married | 4,503 | 18.7 | 24.0 | 16.5 | 21.8 | 21.3 | 36.4 | 30.4 | 35.5 | 19.4 | 22.7 |
|  |  |  | [17.6-19.8] | [22.8-25.2] | [15.4-17.6] | [20.6-23.0] | [20.1-22.5] | [35.0-37.8] | [29.1-31.7] | [34.1-36.9] | [18.2-20.6] | [21.5-23.9] |
|  | Single | 297 | 18.6 | 26.2 | 18.3 | 30.1 | 28.2 | 37.1 | 29.2 | 42.5 | 19.3 | 25.8 |
|  |  |  | [14.2-23.0] | [21.2-31.2] | [13.9-22.7] | [24.9-35.3] | [23.1-33.3] | [31.6-42.6] | [24.0-34.4] | [36.9-48.1] | [14.8-23.8] | [20.8-30.8] |
| Respondent's educational level | Undergraduate or more | 2,551 | 17.1 | 22.5 | 15.8 | 22.1 | 20.6 | 35.8 | 29.1 | 36.7 | 19.4 | 23.4 |
|  |  |  | [15.6-18.6] | [20.9-24.1] | [14.4-17.2] | [20.5-23.7] | [19.0-22.2] | [33.9-37.7] | [27.3-30.9] | [34.8-38.6] | [17.9-20.9] | [21.8-25.0] |
|  | Other | 2,249 | 20.6 | 26.0 | 17.6 | 22.6 | 23.0 | 37.2 | 31.7 | 35.2 | 19.4 | 22.3 |
|  |  |  | [18.9-22.3] | [24.2-27.8] | [16.0-19.2] | [20.9-24.3] | [21.3-24.7] | [35.2-39.2] | [29.8-33.6] | [33.2-37.2] | [17.8-21.0] | [20.6-24.0] |
| Respondent's employment status | Full-time  (tenured) | 2,764 | 18.1 | 22.7 | 17.4 | 23.3 | 23.4 | 38.4 | 30.8 | 38.5 | 19.5 | 24.0 |
|  |  |  | [16.7-19.5] | [21.1-24.3] | [16.0-18.8] | [21.7-24.9] | [21.8-25.0] | [36.6-40.2] | [29.1-32.5] | [36.7-40.3] | [18.0-21.0] | [22.4-25.6] |
|  | Other | 2,035 | 19.6 | 26.1 | 15.6 | 21.0 | 19.6 | 33.8 | 29.8 | 32.5 | 19.3 | 21.3 |
|  |  |  | [17.9-21.3] | [24.2-28.0] | [14.0-17.2] | [19.2-22.8] | [17.9-21.3] | [31.7-35.9] | [27.8-31.8] | [30.5-34.5] | [17.6-21.0] | [19.5-23.1] |
| Partner's educational level | Undergraduate or more | 2,034 | 19.6 | 22.9 | 15.5 | 22.3 | 19.0 | 34.7 | 30.5 | 36.2 | 19.0 | 23.7 |
|  |  |  | [17.9-21.3] | [21.1-24.7] | [13.9-17.1] | [20.5-24.1] | [17.3-20.7] | [32.6-36.8] | [28.5-32.5] | [34.1-38.3] | [17.3-20.7] | [21.9-25.5] |
|  | Other | 2,766 | 18.1 | 25.0 | 17.5 | 22.4 | 23.8 | 37.7 | 30.2 | 35.8 | 19.7 | 22.3 |
|  |  |  | [16.7-19.5] | [23.4-26.6] | [16.1-18.9] | [20.8-24.0] | [22.2-25.4] | [35.9-39.5] | [28.5-31.9] | [34.0-37.6] | [18.2-21.2] | [20.7-23.9] |
| Partner's employment status | Full-time (tenured) | 2,233 | 20.0 | 24.6 | 16.5 | 22.3 | 18.2 | 34.8 | 30.1 | 35.2 | 18.4 | 23.7 |
|  |  |  | [18.3-21.7] | [22.8-26.4] | [15.0-18.0] | [20.6-24.0] | [16.6-19.8] | [32.8-36.8] | [28.2-32.0] | [33.2-37.2] | [16.8-20.0] | [21.9-25.5] |
|  | Other | 2,566 | 17.7 | 23.7 | 16.8 | 22.4 | 24.8 | 37.9 | 30.5 | 36.6 | 20.3 | 22.1 |
|  |  |  | [16.2-19.2] | [22.1-25.3] | [15.4-18.2] | [20.8-24.0] | [23.1-26.5] | [36.0-39.8] | [28.7-32.3] | [34.7-38.5] | [18.7-21.9] | [20.5-23.7] |
| Number of siblings | 0 | 1,187 | 16.5 | 22.8 | 14.5 | 21.9 | 21.8 | 35.7 | 32.6 | 40.8 | 20.1 | 25.6 |
|  |  |  | [14.4-18.6] | [20.4-25.2] | [12.5-16.5] | [19.5-24.3] | [19.5-24.1] | [33.0-38.4] | [29.9-35.3] | [38.0-43.6] | [17.8-22.4] | [23.1-28.1] |
|  | 1 | 3,343 | 19.4 | 25.0 | 17.0 | 22.1 | 22.0 | 36.9 | 29.1 | 34.3 | 19.1 | 21.6 |
|  |  |  | [18.1-20.7] | [23.5-26.5] | [15.7-18.3] | [20.7-23.5] | [20.6-23.4] | [35.3-38.5] | [27.6-30.6] | [32.7-35.9] | [17.8-20.4] | [20.2-23.0] |
|  | 2 or more | 270 | 20.7 | 18.9 | 22.1 | 26.8 | 19.4 | 33.4 | 35.7 | 35.6 | 20.2 | 27.2 |
|  |  |  | [15.9-25.5] | [14.2-23.6] | [17.2-27.0] | [21.5-32.1] | [14.7-24.1] | [27.8-39.0] | [30.0-41.4] | [29.9-41.3] | [15.4-25.0] | [21.9-32.5] |
| Rural-urban status | Rural | 567 | 18.9 | 27.2 | 14.5 | 23.7 | 16.2 | 36.5 | 30.1 | 33.1 | 15.3 | 21.1 |
|  |  |  | [15.7-22.1] | [23.5-30.9] | [11.6-17.4] | [20.2-27.2] | [13.2-19.2] | [32.5-40.5] | [26.3-33.9] | [29.2-37.0] | [12.3-18.3] | [17.7-24.5] |
|  | Suburban | 2,440 | 19.0 | 23.8 | 17.4 | 21.4 | 21.8 | 36.4 | 30.3 | 34.7 | 20.3 | 24.4 |
|  |  |  | [17.4-20.6] | [22.1-25.5] | [15.9-18.9] | [19.8-23.0] | [20.2-23.4] | [34.5-38.3] | [28.5-32.1] | [32.8-36.6] | [18.7-21.9] | [22.7-26.1] |
|  | Urban | 1,793 | 18.4 | 23.5 | 16.3 | 23.2 | 23.5 | 36.5 | 30.5 | 38.6 | 19.5 | 21.3 |
|  |  |  | [16.6-20.2] | [21.5-25.5] | [14.6-18.0] | [21.2-25.2] | [21.5-25.5] | [34.3-38.7] | [28.4-32.6] | [36.3-40.9] | [17.7-21.3] | [19.4-23.2] |
| Annual income | Very low (less than 2 mil. JPY) | 165 | 34.9 | 34.8 | 29.5 | 30.9 | 23.2 | 49.7 | 39.9 | 48.9 | 17.8 | 33.0 |
|  |  |  | [27.6-42.2] | [27.5-42.1] | [22.5-36.5] | [23.8-38.0] | [16.8-29.6] | [42.1-57.3] | [32.4-47.4] | [41.3-56.5] | [12.0-23.6] | [25.8-40.2] |
|  | Low (2 to 4 mil. JPY) | 463 | 22.6 | 28.0 | 20.1 | 24.3 | 27.9 | 40.8 | 39.9 | 41.8 | 27.6 | 27.0 |
|  |  |  | [18.8-26.4] | [23.9-32.1] | [16.4-23.8] | [20.4-28.2] | [23.8-32.0] | [36.3-45.3] | [35.4-44.4] | [37.3-46.3] | [23.5-31.7] | [23.0-31.0] |
|  | Middle (4 to 8 mil. JPY) | 2,294 | 17.8 | 24.5 | 16.0 | 22.3 | 21.2 | 36.3 | 28.9 | 35.3 | 18.2 | 22.5 |
|  |  |  | [16.2-19.4] | [22.7-26.3] | [14.5-17.5] | [20.6-24.0] | [19.5-22.9] | [34.3-38.3] | [27.0-30.8] | [33.3-37.3] | [16.6-19.8] | [20.8-24.2] |
|  | High (more than 8 mil. JPY) | 1,877 | 17.5 | 21.7 | 15.4 | 21.1 | 20.8 | 34.4 | 28.8 | 34.2 | 19.0 | 21.4 |
|  |  |  | [15.8-19.2] | [19.8-23.6] | [13.8-17.0] | [19.3-22.9] | [19.0-22.6] | [32.3-36.5] | [26.8-30.8] | [32.1-36.3] | [17.2-20.8] | [19.5-23.3] |
| Change in monthly income | Decreased 20% or more | 1,078 | 21.1 | 29.8 | 18.6 | 25.8 | 23.9 | 38.9 | 32.4 | 37.7 | 18.2 | 21.7 |
|  |  |  | [18.7-23.5] | [27.1-32.5] | [16.3-20.9] | [23.2-28.4] | [21.4-26.4] | [36.0-41.8] | [29.6-35.2] | [34.8-40.6] | [15.9-20.5] | [19.2-24.2] |
|  | Decreased less than 20% | 1,039 | 18.4 | 22.3 | 18.3 | 21.2 | 22.1 | 37.2 | 29.7 | 33.3 | 19.2 | 22.2 |
|  |  |  | [16.0-20.8] | [19.8-24.8] | [15.9-20.7] | [18.7-23.7] | [19.6-24.6] | [34.3-40.1] | [26.9-32.5] | [30.4-36.2] | [16.8-21.6] | [19.7-24.7] |
|  | No change | 2,222 | 19.4 | 24.1 | 15.0 | 21.3 | 20.6 | 35.0 | 30.3 | 37.0 | 19.8 | 23.5 |
|  |  |  | [17.8-21.0] | [22.3-25.9] | [13.5-16.5] | [19.6-23.0] | [18.9-22.3] | [33.0-37.0] | [28.4-32.2] | [35.0-39.0] | [18.1-21.5] | [21.7-25.3] |
|  | Increased | 460 | 11.2 | 15.0 | 16.2 | 21.5 | 21.8 | 35.9 | 27.4 | 33.2 | 20.6 | 24.1 |
|  |  |  | [8.3-14.1] | [11.7-18.3] | [12.8-19.6] | [17.7-25.3] | [18.0-25.6] | [31.5-40.3] | [23.3-31.5] | [28.9-37.5] | [16.9-24.3] | [20.2-28.0] |
| Total days of school closure | 30 days or less | 91 | 27.2 | 26.7 | 34.4 | 31.0 | 28.0 | 56.1 | 44.7 | 40.4 | 23.5 | 30.0 |
|  |  |  | [18.1-36.3] | [17.6-35.8] | [24.6-44.2] | [21.5-40.5] | [18.8-37.2] | [45.9-66.3] | [34.5-54.9] | [30.3-50.5] | [14.8-32.2] | [20.6-39.4] |
|  | 31 to 60 days | 708 | 18.5 | 24.7 | 18.3 | 25.1 | 19.3 | 34.4 | 30.4 | 39.8 | 17.4 | 23.3 |
|  |  |  | [15.6-21.4] | [21.5-27.9] | [15.5-21.1] | [21.9-28.3] | [16.4-22.2] | [30.9-37.9] | [27.0-33.8] | [36.2-43.4] | [14.6-20.2] | [20.2-26.4] |
|  | 61 days or more | 4,001 | 18.6 | 23.9 | 15.9 | 21.6 | 22.0 | 36.4 | 30.0 | 35.2 | 19.7 | 22.6 |
|  |  |  | [17.4-19.8] | [22.6-25.2] | [14.8-17.0] | [20.3-22.9] | [20.7-23.3] | [34.9-37.9] | [28.6-31.4] | [33.7-36.7] | [18.5-20.9] | [21.3-23.9] |
| NDD diagnosis | No | 4,359 | 16.7 | 22.1 | 15.2 | 21.0 | 19.1 | 34.2 | 27.7 | 34.4 | 18.4 | 22.1 |
|  |  |  | [15.6-17.8] | [20.9-23.3] | [14.1-16.3] | [19.8-22.2] | [17.9-20.3] | [32.8-35.6] | [26.4-29.0] | [33.0-35.8] | [17.2-19.6] | [20.9-23.3] |
|  | Yes | 441 | 39.0 | 43.6 | 31.3 | 35.8 | 48.1 | 58.4 | 56.8 | 51.8 | 28.9 | 30.7 |
|  |  |  | [34.4-43.6] | [39.0-48.2] | [27.0-35.6] | [31.3-40.3] | [43.4-52.8] | [53.8-63.0] | [52.2-61.4] | [47.1-56.5] | [24.7-33.1] | [26.4-35.0] |
| Total PHQ-9 score at Wave 1 | Less than 15 | 4,700 | 18.1 | 23.5 | 16.2 | 21.8 | 21.2 | 35.9 | 29.8 | 35.6 | 19.4 | 22.8 |
|  |  |  | [17.0-19.2] | [22.3-24.7] | [15.1-17.3] | [20.6-23.0] | [20.0-22.4] | [34.5-37.3] | [28.5-31.1] | [34.2-37.0] | [18.3-20.5] | [21.6-24.0] |
|  | 15 or more | 100 | 50.7 | 52.7 | 35.2 | 45.0 | 49.3 | 60.7 | 55.1 | 54.8 | 17.8 | 24.5 |
|  |  |  | [40.9-60.5] | [42.9-62.5] | [25.8-44.6] | [35.2-54.8] | [39.5-59.1] | [51.1-70.3] | [45.4-64.8] | [45.0-64.6] | [10.3-25.3] | [16.1-32.9] |

*Note:* Weighted value was used. NDD = Neurodevelopmental Disorders; PHQ-9 = 9-item Patient Health Questionnaire; SDQ = Strengths and Difficulties Questionnaire.

**Table S4.** Results of Logistic Regression Analysis Predicting Clinical-Level Emotional/Behavioral Problems at Wave 2.

|  | Emotional Symptoms | | | Conduct Problems | | | Hyperactivity/Inattention | | | Peer Relationship Problems | | | Prosocial Behavior | | |
| --- | --- | --- | --- | --- | --- | --- | --- | --- | --- | --- | --- | --- | --- | --- | --- |
| Explanatory variables | OR [95% CI] | | *p* | OR [95% CI] | | *p* | OR [95% CI] | | *p* | OR [95% CI] | | *p* | OR [95% CI] | | *p* |
| Child's sex | 1.00 | [0.80-1.26] | .981 | 1.08 | [0.86-1.36] | .502 | 1.00 | [0.81-1.24] | .987 | 0.88 | [0.73-1.07] | .193 | 0.90 | [0.72-1.11] | .321 |
| Child's school grade | 0.94 | [0.91-0.98] | .005 | 0.97 | [0.93-1.01] | .140 | 0.91 | [0.87-0.94] | < .001 | 1.01 | [0.97-1.04] | .645 | 1.01 | [0.97-1.05] | .672 |
| Respondent's sex | 0.76 | [0.54-1.07] | .109 | 0.82 | [0.56-1.21] | .322 | 0.80 | [0.59-1.08] | .138 | 0.83 | [0.61-1.13] | .233 | 1.09 | [0.76-1.54] | .648 |
| Respondent's age | 0.98 | [0.95-1.01] | .123 | 0.98 | [0.96-1.01] | .170 | 0.98 | [0.96-1.01] | .114 | 1.00 | [0.98-1.02] | .775 | 1.02 | [0.99-1.04] | .149 |
| Respondent's marital status | 0.90 | [0.56-1.46] | .674 | 0.62 | [0.38-1.00] | .050 | 0.86 | [0.56-1.32] | .496 | 0.72 | [0.48-1.09] | .120 | 0.88 | [0.56-1.37] | .562 |
| Respondent's educational level | 0.98 | [0.76-1.27] | .883 | 1.07 | [0.82-1.41] | .617 | 0.99 | [0.78-1.27] | .940 | 1.09 | [0.87-1.36] | .461 | 1.09 | [0.84-1.40] | .523 |
| Respondent's employment status | 0.91 | [0.67-1.23] | .531 | 1.14 | [0.83-1.57] | .421 | 1.08 | [0.82-1.42] | .597 | 1.29 | [0.99-1.69] | .061 | 1.15 | [0.84-1.56] | .380 |
| Partner's educational level | 0.87 | [0.67-1.12] | .276 | 0.94 | [0.72-1.24] | .677 | 0.87 | [0.69-1.10] | .243 | 1.09 | [0.87-1.36] | .439 | 1.06 | [0.83-1.36] | .636 |
| Partner's employment status | 1.05 | [0.78-1.42] | .751 | 1.09 | [0.82-1.47] | .552 | 1.00 | [0.77-1.30] | .991 | 1.13 | [0.89-1.45] | .315 | 1.21 | [0.90-1.62] | .201 |
| Number of siblings | 0.92 | [0.80-1.05] | .200 | 1.01 | [0.89-1.15] | .852 | 0.93 | [0.83-1.05] | .231 | 0.96 | [0.86-1.06] | .400 | 0.97 | [0.86-1.09] | .574 |
| Rural-urban status | 1.00 | [0.83-1.20] | .972 | 1.12 | [0.93-1.35] | .245 | 0.98 | [0.83-1.15] | .761 | 1.17 | [1.00-1.37] | .057 | 0.94 | [0.79-1.13] | .523 |
| Annual income | 1.00 | [0.95-1.05] | .931 | 1.03 | [0.97-1.09] | .355 | 1.00 | [0.95-1.05] | .967 | 0.96 | [0.92-1.00] | .062 | 0.95 | [0.90-0.99] | .028 |
| Change in monthly income | 0.99 | [0.95-1.03] | .467 | 0.97 | [0.93-1.01] | .156 | 1.00 | [0.96-1.04] | .945 | 1.02 | [0.98-1.06] | .354 | 1.01 | [0.97-1.06] | .517 |
| Total days of school closure | 1.00 | [0.99-1.01] | .762 | 0.99 | [0.99-1.00] | .130 | 0.99 | [0.99-1.00] | .086 | 1.00 | [0.99-1.00] | .209 | 1.00 | [0.99-1.01] | .825 |
| NDD diagnosis | 1.26 | [0.88-1.80] | .219 | 0.90 | [0.61-1.33] | .595 | 1.07 | [0.77-1.49] | .701 | 0.92 | [0.64-1.30] | .621 | 0.99 | [0.68-1.42] | .940 |
| Parental depression at Wave 1 | 1.00 | [0.96-1.03] | .866 | 0.99 | [0.96-1.03] | .637 | 1.01 | [0.98-1.04] | .682 | 0.99 | [0.95-1.02] | .517 | 1.00 | [0.96-1.04] | .923 |
| Parental depression at Wave 2 | 1.11 | [1.09-1.14] | < .001 | 1.10 | [1.07-1.13] | < .001 | 1.07 | [1.05-1.10] | < .001 | 1.07 | [1.04-1.09] | < .001 | 1.00 | [0.98-1.03] | .808 |
| Emotional symptoms at Wave 1 | 1.49 | [1.39-1.59] | < .001 | 0.96 | [0.90-1.03] | .246 | 1.03 | [0.97-1.09] | .374 | 1.02 | [0.97-1.08] | .408 | 1.02 | [0.96-1.08] | .583 |
| Conduct problems at Wave 1 | 1.04 | [0.95-1.13] | .432 | 1.65 | [1.51-1.79] | < .001 | 1.06 | [0.99-1.13] | .092 | 0.95 | [0.88-1.03] | .191 | 1.03 | [0.97-1.11] | .344 |
| Hyperactivity/inattention at Wave 1 | 1.01 | [0.95-1.08] | .769 | 1.10 | [1.03-1.16] | .002 | 1.66 | [1.56-1.77] | < .001 | 1.04 | [0.98-1.10] | .175 | 1.04 | [0.99-1.10] | .139 |
| Peer relationship problems at Wave 1 | 1.05 | [0.98-1.11] | .155 | 1.02 | [0.95-1.09] | .644 | 0.98 | [0.92-1.04] | .502 | 1.83 | [1.71-1.96] | < .001 | 1.04 | [0.98-1.11] | .191 |
| Prosocial behavior at Wave 1 | 0.99 | [0.93-1.04] | .572 | 0.96 | [0.91-1.02] | .172 | 0.97 | [0.92-1.01] | .157 | 0.98 | [0.94-1.03] | .420 | 0.63 | [0.59-0.67] | < .001 |
|  | *R^2^* | *(SE)* | *p* | *R^2^* | *(SE)* | *p* | *R^2^* | *(SE)* | *p* | *R^2^* | *(SE)* | *p* | *R^2^* | *(SE)* | *p* |
|  | 0.35 | (0.03) | < .001 | 0.35 | (0.03) | < .001 | 0.44 | (0.02) | < .001 | 0.35 | (0.02) | < .001 | 0.33 | (0.03) | < .001 |

*Note:* Weighted value was used. NDD = Neurodevelopmental Disorders.

**Figure S2.** Increases in the Proportion of Clinical-Level Hyperactivity/Inattention by Children’s School Grade Level.


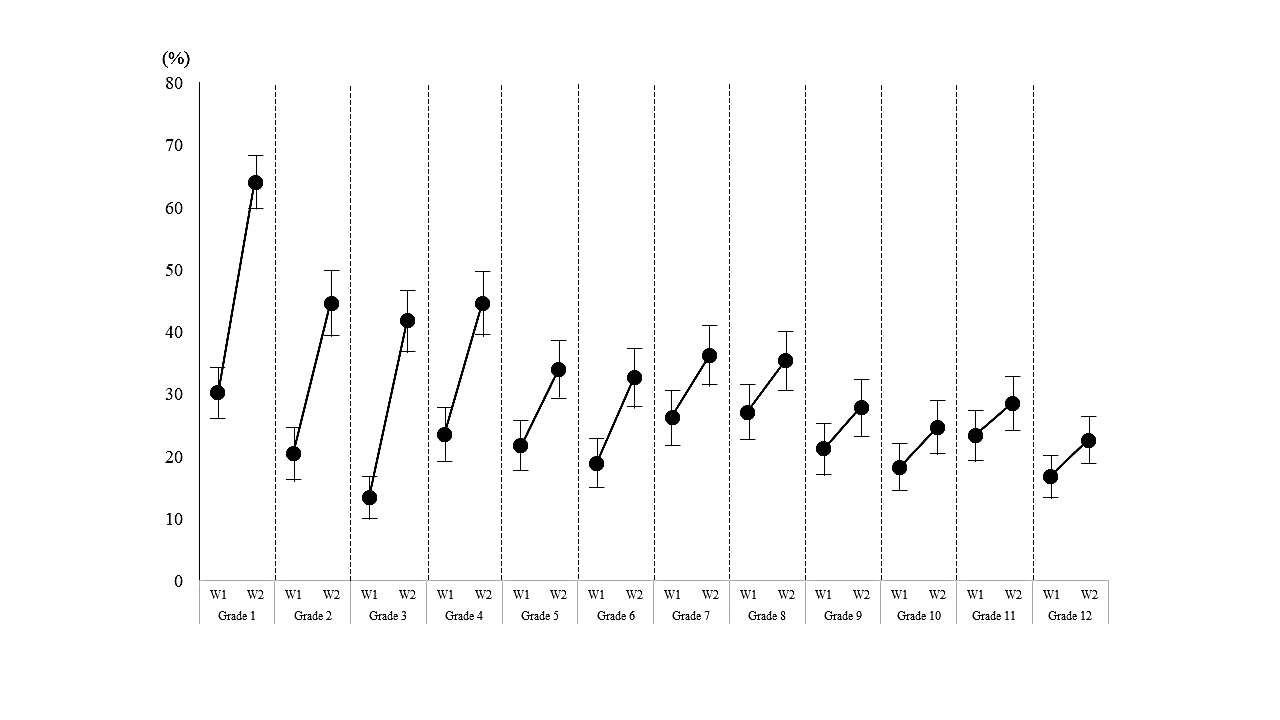


Note: Error bars indicate 95% confidence intervals. W1 = Wave 1. W2 = Wave 2.
